# Community-Based Services for Hospitalized Patients With Serious Injection-Related Infections in Alabama: A Brief Report

**DOI:** 10.1093/ofid/ofae231

**Published:** 2024-04-24

**Authors:** Kelly W Gagnon, William Bradford, John Bassler, Ariann Nassel, Emma Sophia Kay, Madison Jeziorski, Myles Prados, Brandi McCleskey, James Kobie, Ellen Eaton

**Affiliations:** Division of Infectious Diseases, Heersink School of Medicine, University of Alabama at Birmingham, Birmingham, Alabama, USA; Division of Infectious Diseases, Heersink School of Medicine, University of Alabama at Birmingham, Birmingham, Alabama, USA; Center for AIDS Research, Division of Infectious Diseases, University of Alabama at Birmingham, Birmingham, Alabama, USA; Lister Hill Center for Health Policy, University of Alabama at Birmingham, Birmingham, Alabama, USA; Department of Acute, Chronic and Continuing Care, School of Nursing, University of Alabama at Birmingham, Birmingham, Alabama, USA; Division of Infectious Diseases, Heersink School of Medicine, University of Alabama at Birmingham, Birmingham, Alabama, USA; Division of Infectious Diseases, Heersink School of Medicine, University of Alabama at Birmingham, Birmingham, Alabama, USA; Department of Pathology, Heersink School of Medicine, University of Alabama at Birmingham, Birmingham, Alabama, USA; Division of Infectious Diseases, Heersink School of Medicine, University of Alabama at Birmingham, Birmingham, Alabama, USA; Division of Infectious Diseases, Heersink School of Medicine, University of Alabama at Birmingham, Birmingham, Alabama, USA

**Keywords:** geographic disparities, harm reduction, injection drug use, PrEP, US South

## Abstract

Injection-related infections continue to rise, particularly in the South. People who inject drugs are increasingly utilizing hospital services for serious injection-related infections but may be discharged to areas without harm reduction services. We explored the availability and travel time to services for HIV and substance use in Alabama.

## Teaser

Geospatial analysis of community-based harm reduction services for patients hospitalized for a serious injection-related infection in Alabama found an average 16 minute drive time to reach the nearest service site, with few co-located services for infectious diseases and substance use.

In 2021, the southern region of the United States constituted more than half (52%) of all new HIV diagnoses [1]. One in 15 new HIV diagnoses during this time was attributable to injection drug use, 44% of which occurred in the South [1]. People who inject drugs (PWID) are also more likely to contract other infections, such as hepatitis C virus (HCV) and serious injection-related infections (SIRIs) [2–6]. The syndemic of HIV and substance use places PWID at risk of fatal overdose: across international samples, PWID with HIV constitute twice as many overdose deaths as those without HIV [7].

The availability and uptake of harm reduction to prevent and treat HIV and substance use are critical to reduce infection and overdose. Unfortunately, PWID face stigma and barriers to initiating and engaging in prevention services, such as HIV preexposure prophylaxis (PrEP) and behavioral health services [8–13]. PWID increasingly rely on hospital emergency and inpatient services to treat acute injection-related infection [14]. For PWID, these hospitalizations are critical touch points to connect patients to much-needed harm reduction resources in their communities postdischarge to decrease risk of infection and rehospitalization [15–18].

Alabama (AL) has been particularly devastated by the substance use epidemic, with well-characterized hot spots of increasing opioid use disorder (OUD) prevalence in multiple underserved parts of the state [19]. The University of Alabama in Birmingham (UAB) hospital is the only level 1 adult trauma center in AL, resulting in statewide utilization for complex medical needs [20]. Thus, many PWID with SIRI require care at UAB before being discharged to residences across AL where services for HIV, HCV, and substance use may be limited.

This study aimed to explore the geographic distribution of PWID hospitalized at the university hospital for SIRI and their proximity to low-barrier, community-based harm reduction services for HIV, HCV, and substance use. Utilizing secondary data from the electronic health record (EHR) and a survey of service organizations from AIDSVu.org (a patient-facing service database; Emory University), we conducted a geospatial analysis to understand local service availability for PWID who were hospitalized for a SIRI. By identifying the presence of geographic disparities and the types of services available, initiatives can be conducted to improve discharge protocols, including referrals, and community-based service offerings.

## METHODS

### Participant Identification and Characteristics

We expanded on an extant data set, and full details of the methods for data collection and definitions are published elsewhere [21]. Specifically, our inclusion criteria were expanded to include all patients in the sample, regardless of whether they had an OUD, and we extended the study period by a year. Briefly, retrospective data were retrieved from the EHR for patients receiving care for SIRI (skin and soft tissue infections, blood stream infections, bone and joint infections, endocarditis, and brain abscesses) at the UAB university hospital between 11 January 2016 and 24 April 2022. For patients hospitalized for a SIRI, we queried demographics and relevant clinical outcomes. Clinical outcomes included emergency department visit, outpatient visit, patient-directed discharge, and readmission. Chart review was conducted to determine eligibility and whether the patients met clinical criteria for OUD, reported methamphetamine use, received medications for OUD, and left via patient-directed discharge. Patients' self-reported residential ZIP codes were used to determine rurality via urban-centric locale.

### Identification of Harm Reduction Service Organizations

Harm reduction was defined as education, prevention, testing, or treatment services for HIV, HCV, or substance use. We aimed to identify community-based, patient-facing harm reduction service organizations. To achieve this, we utilized the AIDSVu website to search for testing, PrEP, care, overdose prevention, and harm reduction services [22, 23]. We extracted the name, address, and phone numbers of organizations that were generated. To confirm the offering and availability of services, we conducted a brief telephone assessment of each AIDSVu-identified site located in AL. Between August 2022 and January 2023, 2 research assistants called sites to inquire about service availability for people without insurance.

We differentiated services offered by HIV, HCV, substance use, and harm reduction strategies. The HIV category included prescribing PrEP, testing and treatment of HIV, and educational materials. The HCV category comprised HCV testing and treatment. The substance use category consisted of treatment, which could include medications for OUD and/or behavioral health services. Last, the harm reduction strategies category included distributing fentanyl testing strips and/or naloxone.

### Analyses

Address data for locations that provide harm reduction service sites were geocoded with ArcGIS Pro version 3.1 (Esri). The ZIP code associated with the residential address of each patient was merged with its corresponding ZIP Code Tabulation Area (ZCTA), and the population-weighted centroid was calculated for each ZCTA included in the analysis. The driving time between each centroid and the closest harm reduction service location was calculated by the “find closest” tool in ArcGIS Online. Analysis settings for the tool were specified as follows: measurement type = “driving time,” departure time = “time unspecified,” the number of closest locations was limited to 1, and the maximum search range was limited to 120 minutes.

For patient characteristics, clinical outcomes, and harm reduction services, descriptive analyses were conducted and consisted of summarizing available data with measures of central tendency (sample means and medians), dispersion (SD, IQR), and distribution (frequencies, percentages). All analyses were conducted with SAS (version 9.4).

### Patient Consent Statement

This study was approved by the UAB Institutional Review Board and met criteria for exemption. Therefore, written consent from participants was not required.

## RESULTS

### Population Characteristics and Clinical Outcomes

A total of 383 patients met inclusion criteria. Patients were on average 39 years old (SD, 10). A majority were male (59.3%) and White (87.7%) and had residential ZIP codes in rural regions of AL (63.4%; Table 1). Most patients either had public insurance (36.4%) or were uninsured (42.4%). Seventeen percent of patients left the hospital via patient-directed discharge. Only 39.9% had a recorded outpatient visit within 12 months after discharge, while nearly all (97.4%) were readmitted to the hospital and 38.1% visited the emergency department. In total, 64.8% of patients were tested for HIV at admission, and among those tested, 4.4% tested positive (≥200 copies/mL).

**Table 1.** Sociodemographics and Clinical Outcomes of Patients Hospitalized for a Serious Injection-Related Infection at the UAB Hospital and Details of Harm Reduction Services

	No. (%)
**Sociodemographics and clinical outcomes: patients**	383
Age, y, mean (SD)	39.3 (10.4)
Gender: female	156 (40.7)
Race	
White	336 (87.7)
Black or African American	39 (10.2)
Other	8 (2.1)
Insurance plan type	
Private	78 (21.2)
Public	134 (36.4)
Uninsured	156 (42.4)
Missing	15
Urban-centric locale category	
City	70 (18.3)
Suburban	70 (18.3)
Rural	243 (63.4)
Length of stay, d, mean (SD)	21.5 (18.7)
Missing	1
Received medications for OUD at admission	163 (42.6)
HIV test result at admission	
Positive	11 (4.4)
Negative	237 (95.6)
Not tested	158
OUD	296 (77.5)
OUD only	197 (51.6)
OUD and methamphetamine use	99 (25.9)
No OUD	86 (22.5)
Missing	1
Patient-directed discharge	64 (16.8)
Missing	3
Outpatient visit ^a,b^	153 (39.9)
Readmission ^b^	373 (97.4)
Emergency department visit ^b^	146 (38.1)
**Harm reduction services: organizations**	98
HIV testing	75 (77.0)
HCV testing	67 (68.4)
HCV treatment	65 (66.3
Educational materials on HIV	63 (64.3)
Preexposure prophylaxis	61 (62.2)
Substance use treatment	57 (58.2)
HIV treatment	50 (51.0)
Behavioral health for patients with substance use disorders	47 (48.0)
Educational materials on overdose prevention and reversal	29 (30.0)
Medications for OUD	23 (23.5)
Naloxone/Narcan	18 (18.4)
Fentanyl test strips	4 (4.1)
Colocated HIV testing/treatment and substance use treatment/behavioral health	20 (20.4)

Missing data are reported and not included in summary statistics.

Abbreviations: HCV, hepatitis C virus; OUD, opioid use disorder; UAB, University of Alabama in Birmingham.

^a^Outpatient visits included any infectious disease or addiction outpatient clinic visit within the UAB Health System within 12 months of discharge.

^b^Occurs within 12 months of sentinel admission.

### Available Harm Reduction Services and Patient Proximity

A total of 98 qualifying harm reduction service sites were identified from AIDSVu (Table 1). The most commonly offered services were HIV testing (77%), HCV testing (68%), HCV treatment (66%), educational materials on HIV (prevention, testing, and treatment; 64%), and PrEP (62%). The least commonly offered services were medications for OUD (23%), naloxone (18%), and fentanyl testing strips (4%). Twenty harm reduction service sites offered colocated HIV services (testing, PrEP, and treatment) and substance use services (treatment, including behavioral health services).

**Figure 1.**
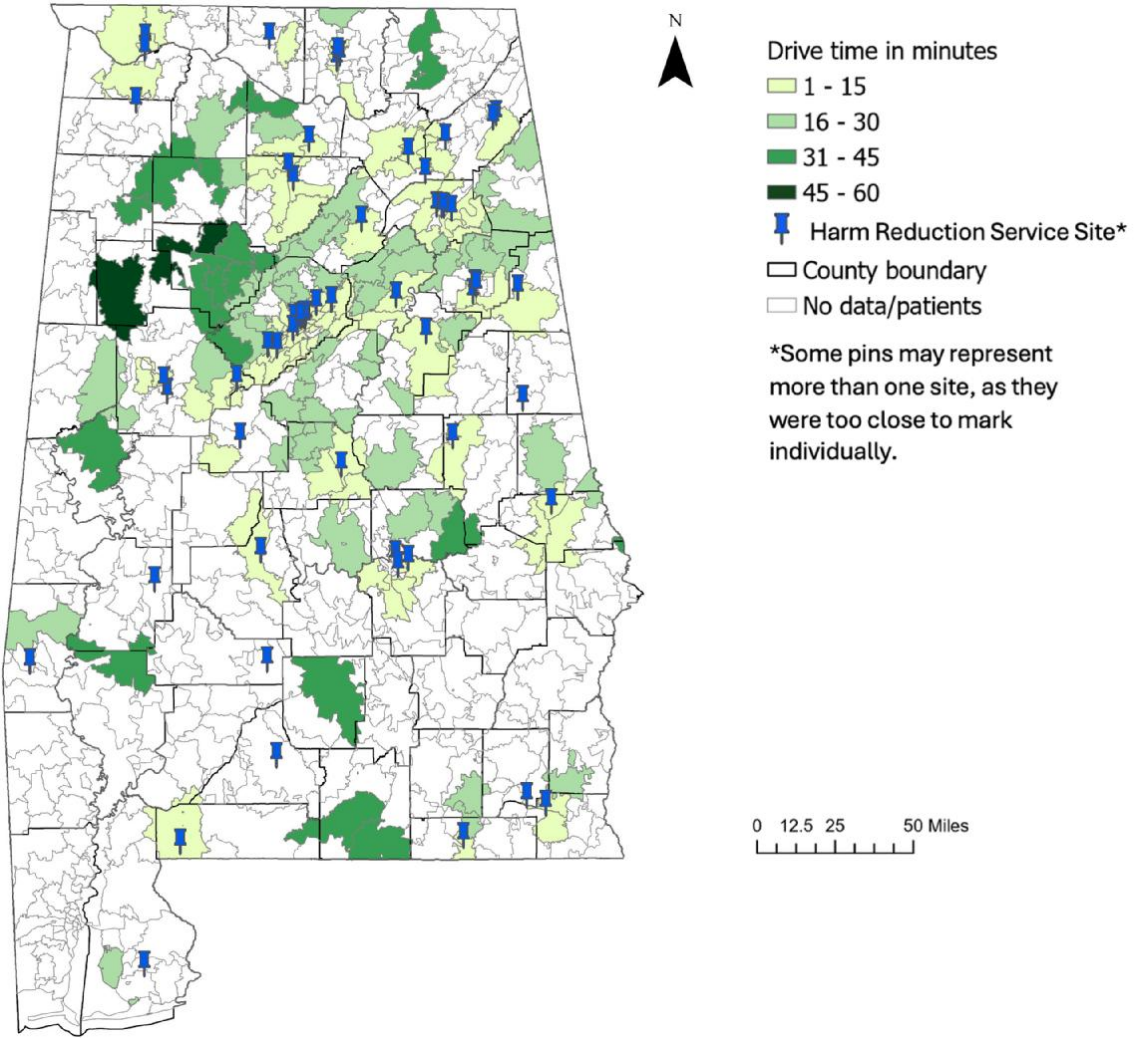
Choropleth Map of drive time to any harm reduction services across population-weighted ZIP Code Tabulation Area for patients hospitalized for a serious injection-related infection at the University of Alabama in Birmingham hospital.

Figure 1 depicts the drive time from the population-weighted center of each ZCTA to the nearest harm reduction service site. On average, it would take 16 minutes from the population-weighted center of ZCTAs to reach the nearest harm reduction service. The 20 harm reduction service sites that offered colocated HIV, HCV, and substance use services were the closest sites for only 31 (17.5%) ZCTAs. To get to these colocated service sites, patients from the 31 ZCTAs had to travel 14.9 minutes on average. Patients from the other 146 ZCTAs would have to travel 17.8 minutes to access their nearest service site, which did not offer colocated services.

## DISCUSSION

This study is the first, to our knowledge, to investigate the distribution of harm reduction services for HIV and substance use in AL for PWID who were hospitalized for a SIRI at the university hospital. This population, like most PWID, experienced many adverse health outcomes following discharge that necessitated additional medical care (eg, readmissions). Yet, community-based services were limited, requiring longer travel and to multiple sites to access comprehensive services.

We found that HIV and HCV harm reduction services were offered at more organizations than substance use services. While there were residential areas near colocated HIV, HCV, and substance use services, the majority of areas and patients living in them did not have colocated services. Distance from harm reduction services is a documented barrier for PWID due to the longer travel times, limited or lack of transportation, and financial constraints [24–27]. Traveling farther decreases patients' ability to stay engaged in outpatient OUD, HIV, and HCV prevention and treatment [27, 28]. This is particularly pronounced in AL, where there is no state funding for public transportation and, consequentially, few or no transportation options for Alabamians without access to a personal vehicle [29].

For tertiary hospitals, which treat many complex SIRI cases, the lack of community-based, colocated services for HIV, HCV, and substance use and the significant travel barriers may exacerbate interruptions to HIV and substance use care continua after discharge. PWID who are treated for SIRI in AL require additional services postdischarge to prevent acute injury. Previous studies have demonstrated the effectiveness of in-hospital dissemination of harm reduction strategies, referrals to existing community-based services, and patient navigation at discharge [17, 30]. While hospital providers are willing to present outpatient treatment options, many are not aware of existing community resources [31].

Previous studies evaluating access to harm reduction to reduce hospitalization among PWID focused on syringe service programs [32, 33]. While these services are associated with decreased hospitalization [32, 33], this harm reduction strategy is not legal in AL. Given current limitations in AL policy, leveraging existing community organizations to distribute naloxone and fentanyl testing strips may be an effective immediate strategy to address OUD and overdose disparities [34, 35]. These community organizations could also utilize mobile services to expand the reach of harm reduction strategies to underserved regions [36, 37].

This study is not without limitations, a few of which have previously been reported [21]. As these data originate from the EHR, we were unable to confirm the ZIP code provided as an area of residence; therefore, it may not be accurate for all patients, such as those who were unstably housed. Additionally, the harm reduction service sites may offer more harm reduction services than what we identified during our service check. However, our survey tool was modeled after patient inquiry; thus, the knowledge of staff answering the phone was used as a surrogate for access. Last, our survey was limited to clinical organizations, as these were the organizations provided by AIDSVu; therefore, service sites such as the state health department were not included.

Our study demonstrates that PWID who were discharged from the hospital for SIRI are often returning to a harm reduction service desert, which acts as a barrier to preventing future injection-related infection and overdose and thus increases risk of readmission. Strategies are needed to improve community-based access, whether through referral at discharge or initiatives to increase service offerings at community-based organizations. Addressing the geographic disparities and lack of colocated services may be the first step to improving access to harm reduction services and ending the substance use, HCV, and HIV syndemic for the most vulnerable PWID in underserved areas.

## Notes

***Data availability*.** Data and materials are available upon request to Ellen Eaton (eeaton@uabmc.edu).

***Financial support*.** This work was supported by the UAB Center for Outcomes and Effectiveness Research and Education (to K. W. G.); the UAB Health Services Foundation Endowment Fund via the School of Medicine at the University of Alabama at Birmingham; the UAB Center for Addiction and Pain Prevention Intervention; the UAB Center for AIDS Research (P30 AI027767); and the National Institute of Mental Health (R25MH083620) (to E. S. K.).
